# Resveratrol increases the sensitivity of breast cancer MDA-MB-231 cell line to cisplatin by regulating intrinsic apoptosis

**DOI:** 10.22038/ijbms.2020.50485.11501

**Published:** 2021-01

**Authors:** Filiz Özdemi̇r, Arda Sever, Yüksel Öğünç Keçeci̇, Zerrin Incesu

**Affiliations:** 1 Department of Biochemistry, Faculty of Pharmacy, Anadolu University, Eskişehir, Turkey

**Keywords:** Apoptosis, Breast cancer, Cisplatin, Combined therapy, Resveratrol

## Abstract

**Objective(s):**

Breast cancer is one of the most common types of cancer. Chemotherapeutic agents used during treatment induce cytotoxic effects also on normal cells in the tissues. Anti-oxidants used in combination with chemotherapeutic agents have been shown to reduce toxicity on normal cells to a minimum, and some anti-oxidant substances have chemotherapeutic effects. Cisplatin (CDDP) is a platinum class drug that is used clinically in the treatment of many cancers. Resveratrol (RSV) is a natural polyphenol with potent anti-oxidant and anticancer properties. In this study, we aimed to investigate apoptotic effects of using cisplatin and RSV alone or in combined treatment of MDA-MB-231 cells.

**Materials and Methods:**

The cytotoxic effects of the drugs on MDA-MB-231 cells were determined by MTT method. Subsequently, the change in CDDP-induced apoptotic effect after RSV addition was examined using the AnnexinV FITC labeling, and TUNEL staining method. Activation of caspase-9, -3 in MDA-MB-231 cells was measured by flow cytometer. The mitochondrial membrane potential (MMP), the major factor on the intrinsic pathway, was measured using flowcytometry.

**Results:**

The combined dose (23 μM CDDP + 72 μM RSV) produced more cytotoxicity than the agents used alone, leading to early apoptosis (8.2%), 31% depolarization, and 23% DNA fragmentation. Caspase-9 was found to be 30.5% in this combined group and caspase-3 was 26.3%.

**Conclusion:**

RSV, an effective anti-oxidant, and CDDP as an effective drug in cancer treatment, were found to increase apoptosis when given in the MDA-MB-231 cell.

## Introduction

Breast cancer, the most common cancer in women, seriously affects the physical and mental health of the afflicted patient (1). Strategies used against breast cancer include surgical removal of the tumor, chemotherapy, radiotherapy and hormone therapy. Despite the presence of many chemotherapeutic drugs today, the ability of cancer cells to develop resistance to these drugs poses a major challenge for the treatment of cancer (2).

Antineoplastic drugs kill cancer cells by stopping them from pathologically growing and multiplying in the body as we as destroying healty cells. Cisplatin (CDDP) is a broad-spectrum period-specific organic chemotherapeutic agent containing a platinum complex. CDDP is an effective antineoplastic drug known to have serious side effects, most seriously peripheral neuropathy and nephrotoxicity. The compound is widely used in the treatment of malignancies, including ovarian, lung, esophagus, stomach, bladder and testicular cancers (3, 4).

Resveratrol (RSV), a natural stilbene and a non-flavonoid polyphenol, is a phytoestrogen that possesses anti-oxidant, cardioprotective, estrogenic/anti-estrogenic, anti-inflammatory, anticoagulant, and preservative effects (5-8). RSV plays a major role in increasing the sensitivity of various cancer cells to chemotherapeutic drugs (9).

The aims of this study are to evaluate the apoptotic effects of RSV on MDA-MB-231 cells, to investigate if its combined use with CDDP can reduce the toxic dose of CDDP and if this combination has an effect on the apoptosis.

## Materials and Methods

***Cell culture and reagents***

The human breast cancer cell line (MDA-MB-231) was obtained from DSMZ (The German Collection of Microorganisms and Cell Culture-Leibniz Institute, Germany). MDA-MB-231 cells cultured in Dulbecco’s modified Eagle’s medium (DMEM) (Sigma, Germany) was supplemented with 10% fetal bovine serum (FBS) (Gibco, UK), and 100 U/ml penicillin/streptomycin (pen/strep). Cells were incubated in a humidified atmosphere containing 5 % CO_2_ at 37 ^°^C. RSV and CDDP were purchased from Sigma-Aldrich (Germany).

***Preparation of concentrations***

Concentrations were prepared in the ranges of 1000, 500, 250, 125, 62.5, 31.25, 15.62, 7.81, 3.90, 1.95, and 0.97 μM to detect IC50 (dose that kills 50% of cells) of CDDP and RSV. The cytotoxic effects of substances on cells were determined by MTT method. For the combination doses, 100%, 75, 50, 40, 30, 20, and 10 percentages of the IC_50_ doses of RSV and CDDP were prepared. The cytotoxic effects of these combinations on the cells were determined by the MTT method.

***Cell viability***

The MTT viability assay was performed by employing slight modifications as previously described (Mosmann, 1983). Briefly, cells (5x10^3^ cells/well) were seeded in 96-well plates and were kept in the incubator for 24 hr. After 24 hr, the concentration range (1000, 500, 250, 125, 62.5, 31.25, 15.62, 7.81, 3.90, 1.95, 0.97 μM) created for CDDP and RSV was added. After 24 hr, 20 μl of MTT dye with a concentration of 5 mg/ml was added to each well in the dark and the plates were kept in the incubator for 3 hr. After 3 hr, the medium and MTT dye were completely withdrawn from the wells, 100 μl of 0.1% DMSO was added to each well to dissolve the formazan crystals, and the plates were kept on a shaker for 15 min. The absorbance was measured at 540 nm using a microplate reader (Bio-Tek, ELX 808 IU). The survival percentage of cells was calculated as follows: cell survival rate (%) = O.D. (experimental value) / O.D. (control value) x 100.

***Flow cytometric analysis of cellular apoptosis***

MDA-MB-231 cells (3x10^5^ cell/ml) were seeded in 25 cm^2^ flask and the cells were treated with either CDDP (IC_50_—46 µM), RSV (IC_50—_144 µM) alone or combination of CDDP + RSV (18.5 µM + 57.5 µM and 23 µM + 72 µM, respectively) for 24 hr. At the end of 24 hr, cells were washed twice with cold PBS followed by removal using 1x trypsin-EDTA. The cells were then transferred to tubes using 1 ml of medium and centrifuged at 1400 rpm for 5 min. After centrifugation, the supernatant in the tubes was decanted and 100 μl of binding buffer was added to each tube and pipetted. Except for the negative control group where no dye was added, 5 μl of FITC-Annexin V and 5 μl of propidium iodide (PI) dye were added to each tube and the tubes were kept in the dark for 15 min at room temperature. After 15 min, another 400 μl of binding buffer was added to the tubes and analyzed on a Becton-Dickinson FACS Aria flow cytometer using FACSDiva Version 6.1.1 software. A total of 10.000 ungated events were acquired for each sample.

***Terminal deoxynucleotidyl transferase deoxyuridine triphosphate (dUTP) nick end labeling (TUNEL) staining for apoptosis***

The fragmented DNA of apoptotic cells was quantified by TUNEL with the *In Situ* Cell Death Detection Kit, Flourescein (Roche). MDA-MB-231 cells were seeded into 24-well plates at 5x10^4^ cells per well and the plates were incubated for 24 hr. At the end of 24 hr, the medium in the wells was completely withdrawn. CDDP, RSV and combinations of these two substances were added to the wells and left to incubate for additional 24 hr. After 24 hr, the medium from the wells was withdrawn and 100 μl of paraformaldehyde solution (4%) was added to each well. After 15 min, paraformaldehyde solution was withdrawn from the wells and each well was washed three times with PBS. After washing, 50 μl of TUNEL reaction mixture was added to each well in a dark environment, the plates were wrapped with parafilm and aluminum foil and kept in the incubator at 37^o^ C for one hour. At the end of one hour, the reaction mixture from each well was withdrawn, and three times washing with PBS was performed under red light. After washing, fluorescently labeled DNA fragments were examined and photographed at 40X magnification using fluorescence microscopy (Leica, Wetzlar, Germany).

***Determination of caspase-9 activity***

MDA-MB-231 cells were incubated with CDDP, RSV and (CDDP + RSV) combination at 37 ° C for 24 hr. At the end of the incubation period, the cells (1 x 10^6^ cell/ml) taken into the culture tube were centrifuged for 3 min at 1200 rpm. Each sample was suspended with 300 μl of cell medium and placed in culture tubes. After adding 1 μl of FITC-LEHD-FMK to the culture tubes, they were incubated at 37°C for 1 hour in an incubator with 5% CO_2_. At the end of the incubation period, samples in the culture tubes were centrifuged at 3000 rpm for 3 min. After the supernatant part was discarded, 500 μl of washing solution was added to each sample and centrifuged at 3000 rpm for 3 min. After the supernatant was discarded, the samples were suspended in 500 μl of washing solution (on ice) and analyzed in flow cytometry (Merck Millipore- FITC-LEHD-FMK)

***Caspase-3 activities assay***

CDDP, RSV and (CDDP+RSV) combination were treated with 5x10^5^ MDA-MB-231 cells with different concentrations for 24 hr and plated. After treatment, caspase-3 activity was measured using the phycoerythrin activated caspase-3 apoptosis kit (BD Biosciences Pharmingen) according to the manufacturer’s instructions. Cell suspensions were centrifuged at 1500 rpm for 5 min and the pellets were washed 2 times with 1.0 ml of cold PBS. The cells were then incubated with 0.5 ml of lysis solution for 30 min at room temperature in the dark. The pellets were washed 2 times with 0.5 ml of washing solution. Cells were resuspended in 100 ml of wash solution, and 10 MI of caspase-3 antibody was added for 20 min at room temperature in the dark. At least 10000 cells were collected for each study. Samples were analyzed in FACSDiva Version 6.1.1 using Becton Dickinson FACS Aria flow cytometer.

***Mitochondrial membrane potential assessment***

Mitochondrial membrane potential (MMP) was assessed using the JC-1 fluorescent probe (BDTM MitoScreen, San Diego, CA, USA) (RUO). In brief, MDA-MB-231 cells were treated with CDDP (46 µM), RSV (144 µM) and a combination of these agents (18.5 µM CDDP + 57.5 µM RSV; 23 µM CDDP + 72 µM RSV) for 24 hr. MMP was assessed using the JC-1 fluorescent probe (BDTM MitoScreen, San Diego, CA, USA) (RUO). At the end of the treatment period, each cell suspension was transferred to a 15 ml polystyrene centrifuge tube and cells were centrifuged at 400 ×g for 5 min at room temperature and supernatant was discarded. 0.5 ml of JC-1 working solution was added to each well and incubated at 37 °C for 15 min in a CO_2_ incubator. After incubation, the cells were washed 2 times by adding assay buffer. Cells were then centrifuged at 400 ×g for 5 min. After centrifugation, each cell pellet was gently resuspended in 0.5 ml of 1 × assay buffer and analyzed on the flow cytometer. Finally, a dot plot of red fluorescence (P1, living cells with intact Δψm) versus blue fluorescence (P2, cells with lost Δψm) was recorded.

***Statistical analysis***

Four wells were used for each group in the MTT experiments and the experiments were repeated 3 times. As with the one way variance analysis (ANOVA), it was aimed to determine the variation among the groups sample, while least significant difference (LSD) test was used in the context of multiple comparasions to determine which group differs. If the *P*-value is 0.05 or lower, the result was accepted as significant.

## Results

***Resveratrol significantly inhibits the proliferation of cancer cells***

**Figure 1:**
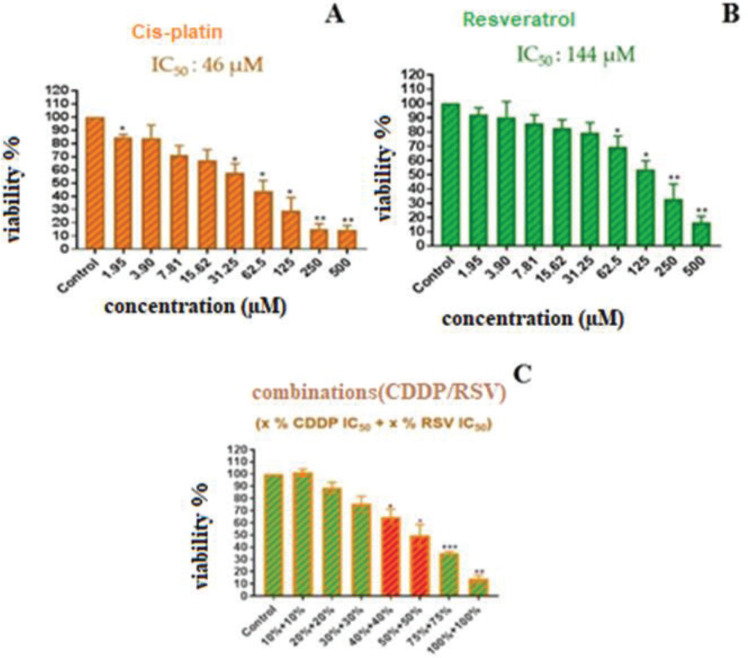
MTT plots of the combination of cisplatin (A), resveratrol (B) and cisplatin/resveratrol (CDDP/RSV) (C) in the MDA-MB-231 cell line after 24 hr. Bars indicate mean±standard deviation. All comparisons were made relative to untreated control cells (100% cell viability). The significant differences were indicated as *P*<0.05 using oneway ANOVA. (*P≤ 0.05, **P≤ 0.01, ***P≤ 0.001)

The IC_50_ values of CDDP and RSV against MDA-MB-231 cells were determined as 46 µM and 144 µM, respectively for 24 hr (Figure 1, A-B). Combination doses were prepared according to IC_50_ doses of CDDP and RSV. The combination of (23 µM CDDP + 72 µM RSV) and (18.5 µM CDDP + 57.5 µM RSV) that are 50% and 40% of IC_50_ values, inhibited cell proliferation by 50.28% and 34.93%, respectively (Figure 1- C, Table 1). The experiments continued by selecting these two combinations.

***Resveratrol induces apoptosis in MDA-MB-231 cells***

**Figure 2:**
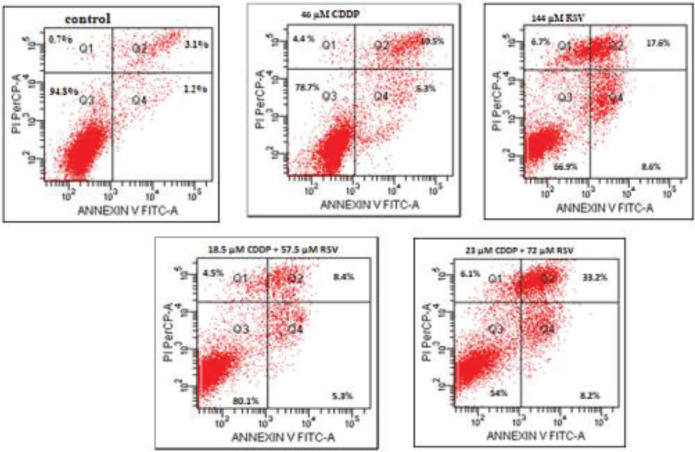
Apoptotic percentage of MDA-MB-231 cells after inducing with cisplatin (CDDP), resveratrol (RSV) and CDDP+RSV in combination for 24 hr. Phosphatidylserine expressing cells were detected by annexin V-binding. Four distinct phenotypes were distinguishable: viable (annexin-V -/PI -, Q3), early apoptotic (annexin-V +/PI -, Q4), late apoptotic (annexinV +/PI +, Q2), and necrotic/damaged cells (annexin-V -/PI +, Q1). Experiments were repeated two times

For quantitative detection of apoptotic effects of alone and combinations of CDDP and RSV on the MDA-MB-231 cell line, the analyses were performed using the flow cytometry. PI and Annexin V-FITC were used to identify viable, apoptotic and necrotic cells in the analysis. Untreated MDA-MB-231 cells were used as control groups. After 24 hr, 46 µM CDDP, 144 µM RSV, and (18.5 µM CDDP + 57.5 µM RSV), (23 µM CDDP + 72 µM RSV) combine groups were analyzed. Early apoptosis was found to be 1.2% in the control group, 6.3% in the group of 46 µM CDDP, 8.6% in the group of 144 µM RSV, 5.3% in the group of (18.5 µM CDDP+57.5 µM RSV), and 8.2% in the group of (23 µM CDDP+72 µM RSV). The results of late apoptosis at these doses are 3.1%, 10.5%, 17.6%, 8.4%, and 33.2%, respectively (Figure 2). Early apoptosis rate in the combination group of (23 µM CDDP+72 µM RSV) was observed higher in the 24 hr duration than 46 μM CDDP, which was applied alone. Similar results were obtained in the case of late apoptosis. Late apoptosis rate was 10.5% in 46 μM CDDP, while 33.2% in the (23 μM CDDP+72 μM RSV) group. Therefore, it was found that when CDDP and RSV were used together, a lower dose of CDDP was required.

***TUNEL assessment***

**Figure 3:**
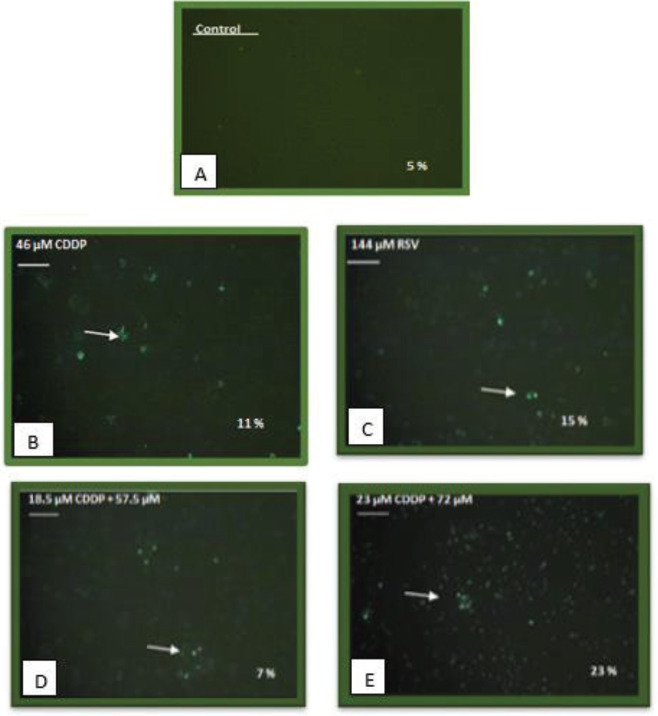
MDA-MB-231 cells were treated with 46 µM Cisplatin (CDDP), 144 µM Resveratrol (RSV), 18.5 µM CDDP + 57.5 µM RSV and 23 µM CDDP + 72 µM RSV for 24 hr. Non-treated MDA-MB-231 cells used as control group and thus gave TUNEL-negative results indicating no apoptotic signal. Arrows indicated cells with fragmented DNA because of which occured actively at the treatment and presence of apoptotic bodies at 24 hr (Magnification X 40)

The “TUNEL” method that is the abbreviation for “TdT-dUTP nick end-labelling”, allows DNA fractures to be recognized *in situ* by marking DNA tips using terminal deoxynucleotide transferase (TdT) due to the apoptotic disruption of cells. Therefore, it is used to separate apoptotic cells from others. The formation of DNA fragmentation in MDA-MB-231 cells incubated with CDDP, RSV and combined drugs for 24 hr was determined with TUNEL method. MDA-MB-231 cell line without any treatment was used as a control group. In the untreated control group, no DNA fragmentation was observed (Figure 3A); however, DNA fragmentation was observed after the treatment of MDA-MB-231 cells with 46 µM CDDP (11%) (Figure 3B), 144 µM RSV (15%) (Figure 3C) and combined (18.5 µM CDDP + 57.5 µM RSV) (7%) and (23 µM CDDP + 72 µM RSV) (23%) (Figure 3D-E). The percentage of apoptotic TUNEL was found to be highest at 23 µM CDDP + 72 µM RSV doses (23%) (Figure 3E).

***Caspase-9 and caspase-3 activity levels***

Caspases are enzymes of the cysteine-protease group that play an important role during apoptosis and have a key role in the elimination of cancer cells. In this study, caspase-9, and – 3 activities were measured after incubation with 46 µM CDDP, 144 µM RSV and combination of both for 24 hr. It was determined that 30.5% of cells treated with the combination of (23 µM CDDP+72 µM RSV) showed activation of caspase-9 after 24 hr. This ratio is higher than when incubated for 24 hr with 46 µM CDDP (8.9%) and 144 µM RSV (12.7%) treated alone. This increase in caspase-9 activation suggests that the use of the anti-oxidant RSV in combination with the anticancer drug CDDP is more effective (Table 2). MDA-MB-231 cells treated with 46 µM CDDP, 144 µM RSV and both combination for 24 hr were examined for determination of caspase-3 activity. After 24 hr incubation, in (23 µM CDDP+72 µM RSV) combination group, caspase-3 activation was found to be maximal in MDA-MB-231 cells (26.3 %). The caspase-3 activations of 46 µM CDDP and 144 µM RSV used alone were 11.7% and 12.4%, respectively (Table 2).

***Mitochondrial membrane potential*** ***changes of*** ***CDDP*** ***, RSV and the combination of (*** ***CDDP*** ***+ RSV) in the MDA-MB-231 cell line***

**Figure 4.**
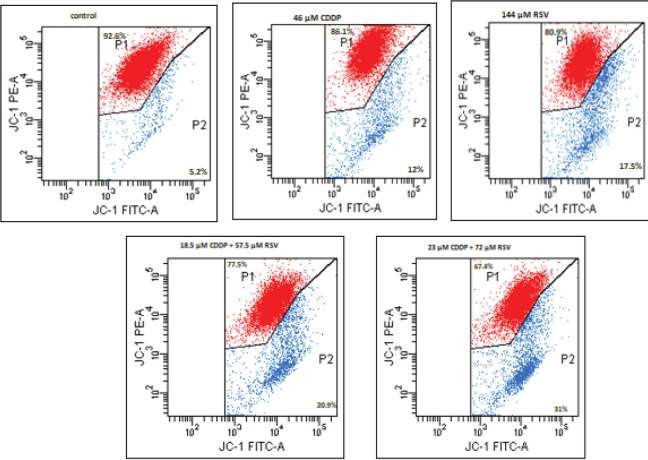
Mitochondrial membrane permeabilization (MMP) of 46 µM cisplatin (CDDP), 144 µM resveratrol (RSV), 18.5 µM CDDP + 57.5 µM RSV, and 23 µM CDDP + 72 µM RSV in MDA-MB-231 breast cancer cells for 24 hr. MMP was determined by flowcytometry using JC-1. The percentages of cells with MMP (blue) were calculated. Gates defined as nonapoptotic, healthy cells (P1), apoptotic cells (P2)

When the percentage changes in MMP of cells undergoing apoptosis caused by CDDP and RSV agents were compared with the control groups after 24 hr, it was found that 12% depolarization occurred at a dose of 46 μM CDDP and 17.5% depolarization at 144 μM RSV. However, in combined doses (23 μM CDDP + 72 μM RSV), the MMP was found to be 31%. We observed that the combined use of (23 μM CDDP + 72 μM RSV) resulted in greater depolarization compared to high-dose 46 μM CDDP (12%) and 144 μM RSV (17.5%) (Figure 4). At the same time, we determined that the combined use of RSV and CDDP significantly reduced the dose of CDDP administered alone.

## Discussion

Cancer that is characterized by uncontrolled growth of cells in the body is considered as an important human health problem, and mortality related to this disease is constantly increasing. Currently, many cancers have gained resistance to the drugs and used cancer treatment regimens have many serious side effects (10). Due to the development of resistance to the drugs in chemotherapy treatment, the current treatments are shifting towards combined medicines. The purpose of combined treatment is to suppress multiple targets at the same time or to regulate various signaling processes responsible for the survival of the tumor through their activation. This is also valid for phytotherapy, where plant extracts contain the amount of bioactive compounds that can have synergistic effects for cancer treatment. Since cancer is a complex disease characterized by changes in dynamic and complex signal pathways that modulate cell growth, survival, differentiation and infestation, the richness of components in the plant extract can affect or target different receptors, so that it can improve the therapeutic effect (11). Many successful anticancer medicines that are still under clinical investigation come from natural products (12), and interests in the use of natural compounds in cancer treatment are growing (13). The studies show that the combined medicines used in cancer treatment have high efficacy and potential. Dietary compounds such as RSV, genistein, curcumin etc. have been recognized as cancer chemo-preventive agents due to the cancer-preventing activities. These compounds also provide antitumor activities by regulating various signaling pathways. Therefore, cytotoxic therapies combined with these dietary compounds may exhibit enhanced synergistic antitumor effects and may also reduce systemic toxicity (14, 15). It has been shown that RSV restricts cell proliferation and adhesion, induces apoptosis, modulates pathways related to angiogenesis and drug resistance, and makes them susceptible to other chemotherapeutics (16, 14). An effective and safe anticancer agent should selectively kill cancer cells without causing too much damage to normal cells. Such an effect can be achieved by the inducing of apoptosis (10). Apoptosis is the balance between cell proliferation and cell death. The distinctive feature of cancer cells is the irregularity between cell proliferation and apoptosis (17). 

The mechanism of apoptosis is known to occur through pathways in which many molecules act together and interact with each other (18). In our study, we found that early and late apoptosis rates were higher in RSV used in combination with CDDP compared to CDDP and RSV applied alone. These results showed that combined use reduces the cytotoxic dose of CDDP by half, resulting in a much more apoptosis than administration alone. Li *et al.* evaluated the anticancer effects of RSV combined with CDDP on small cell lung cancer cells and found that the combination of CDDP+RSV increased the number of apoptotic cells in H446 cells and was significantly more effective in inhibiting H446 cells compared to both monotherapies (19).

The apoptosis process is generally characterized by different morphological features and biochemical mechanisms. Many methods have been designed to detect these changes. DNA fragmentation from these methods can be shown through biochemical (agarose gel electrophoresis) and histochemical (TUNEL) methods. In our study, CDDP, an antineoplastic drug, and RSV, with proven anti-oxidant activity, were applied alone and in combination against MDA-MB-231 cell line for 24 hr. No apoptotic cells were observed in the control group of the MDA-MB-231 cell line (without treatment) compared to the CDDP and RSV groups administered separately. However, a much higher apoptotic effect was found in the CDDP+RSV (23%) group administered as a lower combined dose. Özdemir *et al.* applied Ɛ-viniferin, a derivative of RSV and CDDP, to C6 glioma cells singly and in combination, and compared with the control group, so apoptotic index percentages were found to be 40%, 37.2% and 6.1%, respectively. This rate was found as 41% in the combined group with low dose application (20). To investigate the morphological changes in cells, Lijie *et al.* conducted a study on non-small lung cancer cells (H838 and H520 cells), in which CDDP and RSV were applied in combination and reported that the combined application of CDDP and RSV resulted in much more significant morphological changes in H838 and H520 cells compared to CDDP alone. The authors noted that RSV increases the proliferation inhibition activity of CDDP and its effects of stimulating apoptosis, and they showed this morphologically (21).

Caspases that are synthesized primarily as inactive proteins are enzymes of the cysteine protease group. They are made up of multigene family members that play important roles during apoptotic cell death and are activated in a variety of ways (22, 23). In this study, the roles of caspase-3 and caspase-9, which are parts of the caspase cascade system, were examined. Caspase-9 was 30.5% active in the CDDP+RSV combination group after 24 hr incubation. This rate was higher than the single use of CDDP and RSV. This result shows that RSV combined with CDDP is also effective in increasing caspase-9 activation. Caspase-3 activation was found to be maximum 26.3% in the (CDDP+RSV) combination group when compared to CDDP and RSV used alone. In line with our study, many studies have reported that RSV-induced apoptosis is associated with the activation of the effector caspase-3 and that through activation of caspase-9, the mitochondrial membrane breaks down to result in apoptosis (24, 25). A study on human glioblastoma multiforme T98G cells also evaluated the effect of quercetin that is an anti-oxidant, and temozolomide, which is used to treat brain tumors, singly and in combination on the induction of apoptosis and autophagy, and findings showed that the combination of these two drugs significantly increased caspase-3 and 9 activity (26).

Loss of MMP (ΔΨm) leads to the release of proapoptotic factors into the cytoplasm followed by the continuation of the apoptotic process with cascade of caspase activation. There are numerous studies evaluating MMP using flow cytometry. In our study, we analyzed the integrity of the mitochondrial membrane using JC-1, which is a cationic dye. The JC-1 dye turns blue as an indicator of impaired ΔΨm in apoptotic cells. The MMP is only observed when interruption, apoptosis, or necrosis occur in the cell cycle. Depolarized mitochondrial membrane often indicates that the cell is in the process of early apoptosis. In our study, the highest percentage of depolarization (23 µM CDDP + 72 µM RSV) was found to be in the combine group when compared to 46 µM CDDP and 144 µM RSV groups applied alone. As can be observed from these results, the depolarization percentage of (23 µM CDDP+72 µM RES) (31%) application group was found to be higher than those groups used alone. In consistent with our study, Bobermin *et al.* reported that RSV inhibited the reduction in MMP in astroglial cells (27). In addition, in a study that used viniferin, derived from RSV and is known to have antiproliferative and apoptotic effects, on HepG2 cells, 98.3 µM of ε-VNF, 52.5 µM of vincristine and the combination of (11.25 µM vincristine+15.8 µM ε-VNF) was used and compared to the control group and the ΔΨm was reported 19.5%, 5.5%, 24.6%, 3.5%, respectively. These findings showed that the combination of vincristin+ε-VNF (24.6%) resulted in the early apoptosis of cell in 6 hr (28).

**Table 1:** % inhibition of the combination in the MDA-MB-231 cell line Cisplatin+Resveratrol (CDDP + RSV) after 24 hr

**Selected Combination (%)(CDDP + RSV)**	**Dose (CDDP + RSV)**	**Inhibition %**
40 % + 40 %	(18.5 µM + 57.5 µM)	34.93
50 % + 50 %	(23 µM + 72 µM)	50.28

**Table 2:** Apoptotic percentage of caspase-9 and caspase-3 activity of MDA-MB-231 cells after treatment with different concentrations of cisplatin (CDDP), Resveratrol (RSV) and CDDP+RSV after 24 hr

**Compounds**	**Caspase-9**		**Caspase-3**	
	**Viability %**	**Apoptotic %**	**Viability %**	**Apoptotic %**
Control	98.6	1.4	98.9	1.1
46 µM CDDP	89.7	8.9	88.5	11.7
144 µM RSV	87.6	12.7	87.7	12.4
18.5 µM CDDP+57.5 µM RSV	83.8	16.9	91	9.3
23 µM CDDP+72 µM RSV	70.35	**30.5**	74.9	**26.3**

## Conclusion

When CDDP, an effective chemotherapeutic agent, combined with RSV, a potent anti-oxidant, it is found to induce a higher rate of apoptosis on the MDA-MB-231 breast cancer cell line compared to their monotherapy. And also, we observed that the combined treatment of RSV led to decrease in dosage of CDDP treatment used alone. It also showed that when RSV combined with CDDP, it can increase the antitumor effects of CDDP, at least partly through the above mechanisms.
